# Pharmacogenomics of Cardiovascular Drugs for Atherothrombotic, Thromboembolic and Atherosclerotic Risk

**DOI:** 10.3390/genes14112057

**Published:** 2023-11-09

**Authors:** Alfredo Mauriello, Antonia Ascrizzi, Riccardo Molinari, Luigi Falco, Alfredo Caturano, Antonello D’Andrea, Vincenzo Russo

**Affiliations:** 1 Cardiology Unit, Department of Medical Translational Science, University of Campania “Luigi Campania”—Monaldi Hospital, 80126 Naples, Italy; alfredo.mauriello93@libero.it (A.M.); antonia.ascrizzi@gmail.com (A.A.); riccmolinari@gmail.com (R.M.); luigifalco94@libero.it (L.F.); antonellodandrea@libero.it (A.D.); 2 Department of Experimental Medicine, University of Campania Luigi Vanvitelli, 80100 Naples, Italy; alfredo.caturano@unicampania.it; 3 Unit of Cardiology, “Umberto I” Hospital, Nocera Inferiore, 84014 Salerno, Italy

**Keywords:** antiplatelets, anticoagulants, pharmacogenetics, adverse drug reactions, clinical implementation

## Abstract

Purpose of Review: Advances in pharmacogenomics have paved the way for personalized medicine. Cardiovascular diseases still represent the leading cause of mortality in the world. The aim of this review is to summarize the background, rationale, and evidence of pharmacogenomics in cardiovascular medicine, in particular, the use of antiplatelet drugs, anticoagulants, and drugs used for the treatment of dyslipidemia. Recent findings: Randomized clinical trials have supported the role of a genotype-guided approach for antiplatelet therapy in patients with coronary heart disease undergoing percutaneous coronary interventions. Numerous studies demonstrate how the risk of ineffectiveness of new oral anticoagulants and vitamin K anticoagulants is linked to various genetic polymorphisms. Furthermore, there is growing evidence to support the association of some genetic variants and poor adherence to statin therapy, for example, due to the appearance of muscular symptoms. There is evidence for resistance to some drugs for the treatment of dyslipidemia, such as anti-PCSK9. Summary: Pharmacogenomics has the potential to improve patient care by providing the right drug to the right patient and could guide the identification of new drug therapies for cardiovascular disease. This is very important in cardiovascular diseases, which have high morbidity and mortality. The improvement in therapy could be reflected in the reduction of healthcare costs and patient mortality.

## 1. Introduction

Pharmacogenomics is the science of understanding how genetic variability influences drug treatment outcomes. The prevalence of cardiovascular diseases is increasing over time, and heart diseases represent the leading cause of mortality globally [1]. Patients with cardiovascular diseases frequently show multimorbidity, defined as the presence of at least two pathologies, and are in need of multiple medication regimens, often resulting in polypharmacy, which is defined as five or more daily medications. This condition results in an increased risk of drug–drug interactions (DDIs), drug–gene interactions (DGIs) and drug–drug–gene interactions (DDGIs) [1]. Cardiovascular drugs, in particular antiplatelets and anticoagulants, are the main causes of DDIs, DGIs, and DDGIs. Several studies [1,2,3,4,5] and guidelines [6,7,8] focus on DGIs; however, data on DDGIs remain limited.

## 2. Antiplatelet Drugs

### 2.1. Clopidogrel

Clopidogrel is an oral, irreversible, second-generation P2Y12 inhibitor [2]. It was once the regular thienopyridine prescribed with aspirin for dual antiplatelet therapy (DAPT). However, some pharmacokinetic drawbacks emerged which brought its efficacy into question, even before the approval of more potent, third generation P2Y12 inhibitors. Clopidogrel is a prodrug that requires hepatic biotransformation for generation of active metabolites. Nearly 85% of clopidogrel is inactivated by carboxylesterase-1, while the remaining 15% undergoes two oxidative steps catalyzed by liver cytochromes [2,3]. The Cytochrome P450 C19 (CYP2C19) is crucial, since it participates in both steps to form the active metabolite (R-130964). However, polymorphisms of the *CYP2C19* gene are common, and more than 25 alleles have been identified, conferring different grades of enzymatic activity and accounting for inter-patient variability of the antiplatelet effect [4]. CYP2C19*2 and *3 are the most common and the most studied loss-of-function (LOF) alleles. Both involve single nucleotide polymorphisms (SNPs) of exonic regions producing truncated inactive proteins. Heterozygotic and homozygotic patients are classified as intermediate and poor metabolizers, since they have a reduced or a virtually absent enzymatic activity, respectively [4]. Several studies have reported consistent reduced levels of active clopidogrel metabolite and high platelet residual activity (HPR) in *CYP2C19* LOF carriers [5,6,7]. These patients have an established increased risk of ischemic events compared to those with wild-type *CYP2C19*. Early prospective studies evaluated genotype-based strategies to overcome platelet resistance [8,9]. Both a higher clopidogrel dose and the use of a more potent P2Y12 inhibitor have proved effective in reducing HPR. However, conflicting results emerged regarding hard endpoints, such as death, major adverse cardiovascular events (MACE), and bleeding. Two meta-analyses summarized findings from the myriad of investigations focused on clopidogrel-treated patients [10,11]. In the first meta-analysis, the presence of one or two LOF alleles significantly increased the risk of MACE (Hazard Ratio (HR): 1.55; 95% Confidence Interval (CI): 1.11–2.17, and HR: 1.76; 95% CI: 1.24–2.50, respectively) (*p* = 0.002). The following analysis by Holmes et al. failed to demonstrate a significant association between cardiovascular events and CYP2C19 genotype. However, the fact that genotype assessment was not conducted prospectively and treatment choice was not genotype-guided represented substantial limitations. Seminal randomized clinical trials (RCTs) have established the superiority of ticagrelor and prasugrel over clopidogrel [12,13]. Genetic sub-studies, though, have provided further interesting insights. Indeed, ticagrelor significantly reduced the occurrence of primary endpoint in PLATO compared to clopidogrel in patients with LOF SNPs (*p* = 0.038), whereas a non-significant result was obtained when comparing the ticagrelor group with patients taking clopidogrel with wild-type *CYP2C19* (*p* = 0.06) [14]. In the last years, three RCTs have tested the efficacy of a genotype-guided approach in patients undergoing primary percutaneous coronary intervention (PCI) [15,16,17]. The PHARMCLO (Pharmacogenetics of Clopidogrel in Patients With Acute Coronary Syndromes) was an Italian RCT assessing the safety and efficacy of an antiplatelet strategy based on the results of rapid pharmacogenetic testing and clinical variables compared to standard of care (SOC) [15]. Interestingly, not only was CYP2C19*2 evaluated, the ATP binding cassette subfamily B member 1 (ABCB1) genotype was evaluated as well. This latter encodes an efflux pump, P-glycoprotein (P-gp), which has the potential to hinder Clopidogrel intestinal absorption [18]. Additionally, a gain-of-function allele, CYP2C19*17, related to increased bleeding risk, was also tested [19]. The ultimate P2Y12 selection, though, was at the discretion of physicians. Unfortunately, the RCT was halted prematurely due to regulatory issues with the genotyping tool. However, after 12 months, the primary composite outcome (cardiovascular death, myocardial infarction (MI), stroke, major bleeding according to Bleeding Academic Research Consortium (BARC), type 3 to 5) was significantly reduced in the genotype-guided group vs. the SOC group (HR: 0.58; 95% CI: 0.43–0.78; *p* ≤ 0.001). Ischemic events were significantly lower in the genotype-guided group (13% vs. 21.4%) (HR: 0.57; 95% CI: 0.41–0.8; *p* ≤ 0.001). Finally, there was a non-significant trend to less bleeding events in the pharmacogenomic arm (4.2%) vs. (6.8%) in the SOC arm (HR: 0.62; 95% CI: 0.35–1.1; *p* = 0.1). The POPular Genetics (Cost-effectiveness of Genotype Guided Treatment With Antiplatelet Drugs in STEMI Patients: Optimization of Treatment) exclusively evaluated STEMI patients who underwent PCI [1]. It was a European, multisite, open-label, assessor-blinded, non-inferiority RCT. Enrollment was allowed up to 48 h after PCI. The patients were then randomly assigned to receive a P2Y12 inhibitor according to *CYP2C19* genotype or SOC with ticagrelor or prasugrel. Two primary composite endpoints were assessed: first, net adverse clinical event (NACE), including all causes of death, MI, stent thrombosis, stroke, or major bleeding; second, major or minor bleedings. Diversely from the previous RCT, bleedings were defined according to Platelet Inhibition and Patient Outcomes (PLATO) criteria. The experimental approach was non-inferior to SOC in terms of ischemic events (NACE cumulative incidence 5.1% vs. 5.9%, *p* ≤ 0.001), and superior regarding bleeding risk (9.8% vs. 12.5%, HR: 0.78; 95% CI: 0.61–0.98; *p* = 0.04). More recently, the results of TAILOR-PCI (Tailored Antiplatelet Therapy Following PCI), an international open-label RCT, were published [17]. A total of 5302 patients (82% with acute coronary syndrome and 18% with stable coronary artery disease) were recruited and randomly assigned to either a conventional therapy group or a genotype-guided group. Participants in the first group had priorly received clopidogrel, whereas those in the second group were given clopidogrel or a novel P2Y12 inhibitor (either ticagrelor or prasugrel) according to the *CYP2C19* genotype. There was a non-significant trend towards a reduced occurrence of the primary composite endpoint (cardiovascular death, MI, stroke, stent thrombosis, severe recurrent ischemia at 12 months) in the genotype-guided group (HR: 0.66, 95% CI; 0.43–1.02; *p* = 0.06) without differences in safety endpoints (HR: 1.22; 95% CI; 0.60–2.51; *p* = 0.58). Although the primary outcome did not reach statistical significance, the study provided some positive and hypothesis-generating findings. Genotype-guided treatment reduced the incidence of serious cardiovascular events by 34%. Event curves diverged relatively early after randomization and stayed parallel throughout the RCT duration. The genotype-guided strategy was associated with an almost 80% decrease in the primary composite endpoint at 3 months (HR: 0.21; 95% CI, 0.08–0.54; *p* = 0.001), but this benefit was statistically lost over time. Therefore, most of the benefit related to tailored antiplatelet therapy is achieved in the first few months after PCI, which is consistent with previous studies that have shown a shorter DAPT as potential optimal management [20,21]. It is reasonable to assume that the efficacy and safety of DAPT may be maximized beyond a one-size-fits-all strategy by switching patients who are poor metabolizers to novel P2Y12 inhibitors, which can be effective regardless of the *CYP2C19* genotype [22], and switching patients who are efficient metabolizers back to clopidogrel. Nevertheless, experts in the field have recognized the usefulness of genotype-guided strategies without endorsing its use in routine clinical practice. Data from cerebrovascular and peripheral artery diseases (PAD) are accumulated and provide a rationale for genotype approaches across the entire spectrum of ischemic diseases. Initial evidence came from a post-hoc analysis of the CHANCE [23] (Clopidogrel in High-risk Patients With Acute Non-disabling Cerebrovascular Events) study. This was a double-blind, placebo-controlled RCT that demonstrated the superiority of clopidogrel-based DAPT compared to single antiplatelet therapy (SAPT) with aspirin in patients with minor stroke [24]. The primary composite endpoint included ischemic and hemorrhagic stroke and occurred less frequently in the DAPT group (HR: 0.68; 95% CI, 0.57–0.81; *p* ≤ 0.001). However, statistical significance was lost when comparing *CYP2C19* LOF carriers (HR: 0.93; 95% CI, 0.69–1.26; *p* = 0.93) with non-carriers (*p* for interaction = 0.02). A subsequent meta-analysis confirmed the greater risk of ischemic stroke among carriers [25]. Even more limited data are available for PAD, however a recent systematic review of small studies demonstrated worse outcomes for *CYP2C19* LOF patients [26]. Finally, the multicenter, open-label RCT GENPAD (Genotype-guided Strategy for Antithrombotic Treatment in Peripheral Arterial Disease, (https://classic.clinicaltrials.gov/ct2/show/NCT04619927, accessed on 23 October 2023) is ongoing. Patients were randomized to an SOC group or experimental groups according to *CYP2C19* genotype [27]. Interestingly, intermediate metabolizers were assigned a doubled dose of clopidogrel (150 mg), while poor metabolizers received dual pathway inhibition with aspirin and low-dose rivaroxaban (2.5 mg bid). Results are expected in 2024. In conclusion, Clopidogrel should be avoided in patients who are known to be poor or intermediate metabolizers (Table S1) [4].

### 2.2. Ticagrelor and Prasugrel

Pharmacogenomic data on ticagrelor and prasugrel are scant. Most recent studies have been centered on platelet reactivity rather than clinical endpoints. Those that also explored clinical outcomes, though, were not sufficiently powered to identify significant differences. The aforementioned genetic sub-study of PLATO demonstrated ticagrelor efficacy and safety regardless of genetic background [14]. More recently, a two-step genome-wide association study (GWAS) was conducted [28]. The study included nearly a third of the PLATO population and investigated the prediction of outcomes through the evaluation of additional genetic variants. *SLCO1B1*, *CYP3A4*, and *UGT2B7* variants significantly affected both ticagrelor and ARC (its main active metabolite) plasma concentrations. However, these pharmacokinetic findings did not translate to significant disparities in the primary composite outcome. Therefore, despite the relatively limited number of pharmacogenetic studies investigating the clinical outcomes associated with ticagrelor, the existing data indicate that genetic variants do not have any effect on clinical outcomes in patients treated with ticagrelor. Early pharmacogenomic evidence for prasugrel came from genetic sub-studies of the phase 3 RCT “A Comparison of Prasugrel (CS-747) and Clopidogrel in Acute Coronary Syndrome Subjects Who Are to Undergo Percutaneous Coronary Intervention” (TRITON-TIMI 38) [29,30]. Despite assessing several variants, no statistically significant association was observed between SNPs and outcomes among patients receiving prasugrel. Similar findings emerged from a single-center retrospective Japanese study on patients who had PCI [31]. Therefore, unlike clopidogrel, clinical results are unaffected by genetic variations in many candidate genes. As for ticagrelor, these results agree with current recommendations in both clinical practice guidelines and prescription labeling.

### 2.3. Acetylsalicylic Acid

Acetylsalicylic acid (ASA), better known as Aspirin, irreversibly inhibits cyclooxygenase (COX)-1 and COX-2 activity, acetylating serine 529 and lysine 512, respectively. As a result, synthesis of thromboxane-A2 (TXA2) from arachidonic acid is limited and platelet aggregation is impaired [32].

Despite the well-known beneficial effects of ASA, a significant variability in individual response to this antiplatelet medication has been recognized. Indeed, the development of aspirin resistance is a commonly reported phenomenon [33]. However, its underlying mechanisms are not well understood. The etiology is believed to be multifactorial, with genetic factors playing a predominant role. Multiple genetic association studies, including both genome-wide investigations and candidate gene analyses, have shown the presence of multiple SNPs within genes associated with COX, TXA2, and platelet receptors [34,35]. These SNPs have been found to have a detrimental impact on the efficacy of aspirin. Approximately, a wide range of individuals who take ASA demonstrate a sub-optimal response with different degrees of HPR. As a result, this subset of patients exhibits higher rates of recurrent thromboembolic vascular events. This phenomenon is often referred to as aspirin resistance [36]. Lack of consensus and ambiguous definition, though, resulted in an inconsistent reporting. The European Society of Cardiology (ESC) Working Group on Thrombosis has developed a classification system that distinguished clinical resistance and laboratory resistance [37]. The inability to avert cardiovascular events in patients on ASA therapy is acknowledged as clinical aspirin resistance. These patients may only be retrospectively recognized, since the occurrence of thrombotic events must take place after aspirin initiation. Conversely, laboratory aspirin resistance occurs when in vitro platelet reactivity remains unimpeded despite ASA exposition. This phenomenon arises when there is inadequate suppression of TXA2 synthesis or platelet aggregation. Previous studies have suggested a link between ASA antiplatelet effects and variants in genes encoding COX, TXA2, and platelet surface receptors [37]. However, compared to the amount of data available for clopidogrel, research supporting the translation of those effects into clinical outcomes is still in a more preliminary phase. Considering the hemostatic landscape, several candidate genes have been investigated. However, results were never univocal [38]. PTGS1 and PTGS2 encoding for COX-1 and COX-2, respectively, have been thoroughly studied. Plenty of studies supported the interaction between *rs10306114/rs3842787* with impaired aspirin response; however, the data were inconsistent [39,40]. PTSG2 is a highly polymorphic gene, though only few SNPs affect enzyme function and expression [41,42]. The *rs20417 C* allele was significantly related to aspirin resistance in 450 patients with ischemic stroke (adjusted odds ratio (aOR): 1.75; 95% CI: 1.06–2.88; *p* = 0.016; aOR: 3.16; 95% CI: 1.24–8.03 for Guanyne–Cytosine (GC) and CC genotype patients, respectively). However, it is important to note that not all research supports or confirms these results [43].

Variations in the ITGB3 gene, which encodes for glycoprotein GP IIb/IIIa, the specific fibrinogen receptor, have been linked to varying degrees of aspirin sensitivity, resulting in higher rates of thrombotic events [44].

Szczeklik et al. demonstrated greater aspirin resistance in carriers of the PIA2 allele compared to those carrying the wild type allele (*p* = 0.001) [44]. A comprehensive systematic review, including ten studies that assessed the rs5918 SNP, revealed a significant correlation between the PIA2 allele and in healthy adults (OR: 2.36; 95% CI: 1.24–4.48; *p* = 0.009) [45]. Four studies included healthy participants, whereas six studies involved patients with a cardiovascular disease. The effective size was lost when combining participants with cardiovascular disease and those without it. This observation might be attributed to the possibility that patients with CVD may be using medication that could impact platelet function. Once again, inconsistent results emerged from other studies [46]. Earlier investigations showed an increased platelet aggregation to different agonists despite ASA exposition in healthy individuals with PEAR1 variants [47]. The impact on cardiovascular outcomes was evaluated by Lewis et al. [48] in a post-hoc analysis of the (Aspirin in Reducing Events in the Elderly) ASPREE study [47]. It was a double-blind, placebo-controlled RCT that allocated a 1:1 patient ratio to either daily 100 mg ASA or placebo. The primary composite endpoint included MACE, ischemic stroke, and bleeding events. No significant interaction emerged between *rs12041331* and any of the outcomes [47]. On the other hand, Li et al. [49] reported a significant association between the aforementioned variant and scores measuring neurological function, such as the National Institutes of Health Stroke Scale, Bartel Index, and Modified Rankin Scale.

Recently, aside from relating genetic variants to aspirin resistance, a potential role of gastrointestinal AEs was studied [50]. However, mechanistic studies and replication of results are needed.

## 3. Lipid-Lowering Drugs

### 3.1. Statins

Statins, known as 3-hydroxy-3-methylglutaryl coenzyme A (HMG-CoA) reductase inhibitors, are widely utilized for their effectiveness in treating hypercholesterolemia and preventing coronary artery disease (CAD) [51]. Considerable variability exists, however, in how individuals respond to statin therapy, encompassing both the lipid-lowering effect and the occurrence of adverse reactions [45]. Several candidate genes have been investigated for their role in influencing different responses to statin therapy.

Statins exert their effects by binding to the catalytic domain of HMGCoA-reductase, which results in the blockage of the enzyme’s access to its natural substrate, HMGCoA [46]. Numerous studies have identified single nucleotide polymorphisms within the HMGCoA-reductase gene that can influence the effectiveness of statins in lowering cholesterol levels [46]. One of the most studied variants, known as *rs3846662*, has been linked to statin responsiveness in certain clinical trials, while other trials have not observed the same association [46]. The impact of this single nucleotide polymorphism (SNP) situated within intron 13 of the HMGCoA-reductase gene has been confirmed to result in an alternative splicing event involving exon 13 of the HMGCoA-reductase gene [47]. This alternative splicing results in the expression of a non-functional protein isoform, often referred to as 13 or hydoxy-3-methylglutaryl-CoA reductase variant 1 (HMGCRv1), which lacks 53 amino acids within the catalytic domain of the enzyme [47]. This region of the enzyme is where statins typically bind to exert their inhibitory effects [47].

The KIF6 kinesin is a member of a family of molecular motors responsible for the intracellular transport of protein complexes. A specific genetic variant within KIF6, known as *rs20455* or *W712R*, was initially identified as an independent risk factor for cardiovascular disease [48]. Following these observations, subsequent research indicated a potential positive correlation between *KIF6 rs20455* and the response to various statins, such as pravastatin and atorvastatin [49]. These early findings resulted in the creation of a commercially available test for KIF6 genotyping. However, more recent studies did not validate those associations. Accordingly, to date, genetic testing for this variant is not recommended [50,51]. Consequently, the role of *KIF6 rs20455* in predicting statin response or CVD risk remains uncertain, and its use in clinical practice is still a subject of ongoing investigation and debate.

Regarding organic anion transporting polypeptide 1B1 (OATP1B1) transporter (gene symbol: *SLCO1B1*), in vitro pharmacokinetic studies have identified numerous polymorphisms, but only two relatively common non-synonymous SNPs, specifically c.388A4G and c.521T4C, have been found to significantly alter the transporter’s function [52].

These single nucleotide polymorphisms are linked to specific haplotypes known as solute carrier organic anion transporter family member B1: SLCO1B1*5 and SLCO1B1*15. Both haplotypes lead to a reduction in transport function. Studies on the pharmacokinetics of statins conducted in vivo provided further evidence to support these findings, suggesting that just one copy of either of these variant haplotypes is sufficient to elevate the concentration of statins in the bloodstream [52]. Furthermore, it has been suggested that this alteration in statin concentration can affect the ability of statins to lower cholesterol levels, a hypothesis that has been confirmed by several small-scale in vivo pharmacodynamic studies [53]. However, larger studies have yielded less definitive results [53,54].

Statins are typically well tolerated, but, in some cases, can lead to myopathies, encompassing a range of symptoms from mild muscle aches (myalgias) to severe, life-threatening rhabdomyolysis. The precise mechanism underlying statin-associated myopathies is still not fully understood, but it seems to be connected to the elevated concentrations of statins [55]. Statin concentrations in the bloodstream are influenced by two primary factors: first-pass uptake into hepatocytes and the rate of metabolism by hepatic cytochromes P450 (CYP450) enzymes (liver enzymes responsible for drug metabolism) [55]. Genetic variations affecting hepatic uptake and statin metabolism have been linked to alterations in statin concentrations and the risk of developing myopathies [56,57]. In addition to the previously described impact on pharmacokinetics, the polymorphisms of OATP1B1 transporter have been linked to statin-induced myopathy. The *SLCO1B1* c.521T>C SNP has been strongly linked to the development of myopathy in individuals taking simvastatin. In a study involving 85 patients who experienced myopathy while being treated with a daily dose of 80 mg of simvastatin, along with 90 control subjects who did not develop myopathy, researchers analyzed approximately 300,000 genome markers, discovering a noncoding SNP within the *SLCO1B1* gene in nearly complete linkage disequilibrium with the c.521T>C SNP, showing a significant association with myopathy at a genome-wide significance level [58,59]. More specifically, it was found that over 60% of the cases of myopathy could be attributed to the presence of the c.521T>C SNP, and individuals carrying one copy of the c.521C allele had an odds ratio of 4.5 for developing myopathy compared to those without this allele [53]. The impact of a previously reported genetic variation in *SLCO1B1* on the risk of Statin-Associated Muscle Symptoms has been incorporated in the latest CPIC 2022 guidelines, with specific recommendations regarding statin intensity and statin dose stratified by *SLCO1B1* (Table S2) [57].

The *ABCG2* gene encodes a transporter known as the adenosine triphosphate (ATP)-binding cassette and is found in various tissues throughout the body. *ABCG2* plays a role in exporting compounds into the extracellular space. A well-studied genetic variant of this gene, p.Q141K (also known as c.421C>A and *rs2231142*), is associated with a 30–40% reduction in protein expression when the minor A allele is present, leading to increased levels of rosuvastatin in the bloodstream. When an individual carries one normal function allele along with one decreased function allele (*rs2231142*; c.421C>A), their *ABCG2* function is diminished. If an individual has two decreased function alleles, their *ABCG2* function is considered poor. The 2022 CPIC Guidelines regarding ABCG2 are primarily related to rosuvastatin. For individuals with poor *ABCG2* function, it is recommended to initiate rosuvastatin treatment at a dose of 20 mg or less. However, if a dose exceeding 20 mg is required to achieve the desired therapeutic effect, an alternative statin or combination therapy, such as a statin in combination with ezetimibe, is suggested as a more suitable option (Table S2) [57]. Significant associations have been found between certain cytochrome 3A (CYP3A) and cytochrome 2C9 (CYP2C9) genetic polymorphisms and the blood concentrations of statins. CYP3A4*22 (*rs35599367*) is a decrease-of-function polymorphism that results in a substantial reduction in CYP3A4 enzyme levels and activity [60]. This genetic variant significantly alters the pharmacokinetics and dynamics of statins such as simvastatin, atorvastatin, and lovastatin [61]. One study found that the bioavailability of simvastatin was nearly 50% higher in individuals carrying the CYP3A422 variant compared to those with the wild-type allele [62]. While the role of cytochrome P450 3A5 (CYP3A5) in the metabolism of statin drugs is not as significant as that of CYP3A4, associations between CYP3A5 polymorphisms and changes in statin pharmacokinetics and dynamics have been observed. Among the various CYP3A5 variants studied, the loss-of-function CYP3A5*3 allele (*rs776746*) is the most extensively investigated. Several studies have shown that simvastatin bioavailability and area under curve (AUC) were significantly higher in individuals carrying the CYP3A5*3 variant compared to wild-type carriers. In a study, the AUC for simvastatin was 2.3- and 3.3-fold higher in individuals with heterozygous and homozygous CYP3A5*3 alleles compared to those with the wild-type allele [59]. Similarly, additional research confirmed these findings by showing that 12 h post-dose concentrations of simvastatin and its metabolites were 20% and 14% higher, respectively, in CYP3A5*3 carriers compared to individuals with the wild-type allele and 33% higher in homozygous CYP3A5*3 carriers compared to those with functional CYP3A5 [63,64,65]. In conclusion, high-dose simvastatin should be avoided in patients known to be homozygous for the SLCO1B1*5 reduce functions variant. CYP2C9 enzyme plays a critical role in the hepatic clearance of fluvastatin. Genetic variations in the *CYP2C9* gene have been linked to substantial alterations in the metabolism of fluvastatin. In one study, healthy volunteers were administered 40 mg of fluvastatin daily for a period of 14 days; individuals possessing the CYP2C9*3/*3 (*rs1057910*) genotype experienced a threefold increase in the area under the curve (AUC) for the active form of fluvastatin compared to those with the CYP2C9*1/*1 wild-type genotype [66,67]. In a different study, patients with CYP2C9*1/*3 genotype, which is linked to reduced enzyme function, achieved a more substantial reduction in both LDL-C (low-density lipoprotein cholesterol) and total cholesterol levels when compared to individuals carrying the fully functional *CYP2C9* gene. This suggests that genetic variations in CYP2C9 can influence the response to fluvastatin treatment, with those having the *1/*3 genotype experiencing a more significant beneficial effect on their cholesterol levels [62]. Another extensively studied variant is CYP2C9*2 (*p.R144C*; *rs1799853*), which reduces CYP2C9 function by 30–40%, leading to increased systemic exposure to fluvastatin. The two CYP2C9 variants described (CYP2C9*2 and CYP2C9*3) were incorporated into the 2022 CPIC guidelines; CYP2C9 poor-metabolizers should not receive more than 20 mg per day as a starting dose, and doses of Fluvastatin should be adjusted (Table S2) [57].

### 3.2. Ezetimibe

Ezetimibe is a cholesterol absorption inhibitor; its molecular target is the Niemann-Pick C1-like 1 (NPC1L1) sterol transporter located on the small intestine’s brush border. This protein is responsible for the intestinal uptake of cholesterol and phytosterols [68,69]. Ezetimibe undergoes a phase II metabolic transformation into its phenolic glucuronide within the intestinal wall. Over 95% of ezetimibe is already glucuronidated in the portal vein [70]. Ezetimibe is then uptaken by the liver and excreted into the bile, subsequently returning to the intestinal lumen where it can effectively inhibit cholesterol absorption. Notably, Ezetimibe is not metabolized by cytochrome P450 enzymes, leading to no significant interactions with most other medications [70]. Ezetimibe glucuronide is a high-affinity substrate for the hepatic uptake carrier OATP1B1, which belongs to the OATP family (gene symbol: *SLCO*) [71]. Hepatic uptake of Ezetimibe and, consequently, the entero-hepatic circulation of its active metabolite, Ezetimibe-glucuronide, can be affected by genetic polymorphisms in OATP1B1. Specifically, the nonsynonymous *SLCO1B1* c388A>G variant appears to influence how the transporter recognizes substrates and, as a result, its function [71]. It is worth noting that while this polymorphism has an impact on the disposition of the drug, it does not significantly alter the pharmacodynamic effects of Ezetimibe [72].

Within the intestine, Ezetimibe primarily undergoes metabolic transformation by UDP glucuronosyltransferase 1 family, polypeptide A1 (UGT1A1) [66]. It is noteworthy that both Ezetimibe and its glucuronide form serve as substrates for the efflux pumps known as ABCB1 and ATP binding cassette subfamily B member 2 (ABCC2). These efflux pumps, along with 1, are susceptible to interactions with other drugs, and such interactions have been observed to affect the effects of Ezetimibe. For instance, when subjects were administered the inducer rifampin, which stimulates ABCB1, ABCC2, and UGT1A1, the clearance of both Ezetimibe and its glucuronide from the intracellular compartment was significantly increased, ultimately nullifying the cholesterol-lowering effects of Ezetimibe [73]. Furthermore, UGT1A1, ABCB1, and ABCC2 exhibit characterized genetic variations in their functionality. Although the precise impact of such genetic variations on the response to Ezetimibe remains unclear, it is theoretically possible that individuals with variants associated with reduced functionality might experience an enhanced response to Ezetimibe, while those with increased functionality could have a diminished response to the drug.

Finally, several *NPC1L1* gene polymorphisms were associated with different responses to Ezetimibe. In a study, one individual who did not respond to Ezetimibe was identified as a compound heterozygote for two rare nonsynonymous polymorphisms in the *NPC1L1* gene [56]. The first polymorphism was found in exon 2, at codon 55, represented as g219t (GTG>TTG), and commonly referred to as V55L. The second polymorphism was located in exon 18, at codon 1233, indicated as t3754a (ATC>AAC) [74]. These specific polymorphisms were not found in the normal control subjects used for comparison [74]. In a study involving 101 individuals, subjects not possessing the common *NPC1L1* haplotype characterized by the sequence 1735C-25342A-27677T experienced a notably more substantial reduction in plasma LDL cholesterol when treated with Ezetimibe compared to subjects who had at least one copy of this common haplotype [75]. In another study, it was noted that individuals possessing the specific haplotype combination of -133 A, -18 A, and 1679 G exhibited a more substantial reduction in LDL cholesterol levels in response to Ezetimibe treatment [76].

### 3.3. Proprotein Convertase Subtilisin/Kexin Type 9 Inhibitors (PCSK9)

PCSK9 inhibitors target a serine protease enzyme critical for regulating cholesterol levels. This enzyme binds to the EGF-A domain of LDL receptors, leading to their degradation [71]. A decrease in LDL receptors reduces the clearance of LDL-C, causing an increase in LDL-C levels [77]. Since the discovery of the *PCSK9* gene, considerable pharmaceutical efforts have focused on PCSK9 inhibition. Monoclonal antibodies designed to inhibit PCSK9 have shown the ability to lower LDL-C levels by up to 57% when used alone and up to 73% when combined with statin medications [78]. While there is abundant information available about mutations in the *PCSK9* gene and their impacts on LDL-C levels, our understanding of the pharmacogenomics of PCSK9 inhibitors remains limited to the present. A case of inadequate response to PCSK9 inhibitors therapy was documented in a patient with familial hypercholesterolemia [79]. Analyzing the patient’s LDL receptor, a heterozygous mutation was detected, specifically the 1448G>A (W483X) mutation. Notably, the patient’s serum levels of PCSK9 (proprotein convertase subtilisin/kexin type 9) were measured at approximately 71.30 ± 26.66 ng/mL, which closely corresponds to the typical levels documented in the medical literature. This genetic mutation in the LDL receptor, along with the observed PCSK9 levels, provides insights into the patient’s condition and its potential implications for their response to treatment. It is suggested that this LDL receptor mutation may have an impact on its metabolism, potentially rendering the response to PCSK9i treatment as ineffective [79]. In another case of familial hypercholesterolemia, a patient was found to have compound heterozygote mutations, specifically *R410S* and *G592E*, in the *LDL receptor* gene [80]. The patient’s response to maximum therapy with rosuvastatin and ezetimibe in combination with a PCSK9i was modest. Further examination of the genetic mutations revealed that the *LDLR-G592E* mutation falls into class 2b, primarily because it caused the LDL receptor to fail in exiting the endoplasmic reticulum, leading to its degradation within the cell. On the other hand, the *LDLR-R410S* mutation did not affect the levels of LDL receptors on the cell surface, nor did it interfere significantly with PCSK9 binding to the cell surface LDL receptor. However, it played a role in preventing the degradation of LDL receptors induced by extracellular PCSK9 in endosomes/lysosomes. This unique genetic combination and interaction between these mutations may provide an explanation for the patient’s resistance to PCSK9 inhibitor treatment, as it hindered the expected response to this therapy [80]. Currently, there are no studies on the association of *PCSK9* gene variants with poor or non-response. Pharmacogenomics applied to PCSK9 inhibitors is a promising therapeutic target for the evaluation of potential genetic factors that may play a role in suboptimal drug response.

### 3.4. Fibric Acid Derivatives (Fibrates)

Fibrates are a class of lipid-lowering drugs used in the treatment of hypertriglyceridemia. Structurally, they are amphipathic carboxylic acids [81]. The intake of fibrates reduces plasma triglyceride levels with a reduction in VLDL and an increase in HDL [82]. These effects are a consequence of gene expression induced by binding of fibrates to some subclasses of PPAR receptors (peroxisome proliferator-activated receptors). PPARs, in their different isoforms, play a key role in adipogenesis, lipid metabolism, insulin sensitivity, inflammation, and blood pressure regulation [83]. Fibrates especially activate the α subclass (PPAR-α) [84], modulating the transcription of key drug target genes containing a PPAR-α response element. Several candidate genes have been investigated for their role in influencing different responses to fibrates therapy. Evidence from candidate gene studies and genome-wide association studies have provided valuable insights into the modulation of fibrate response. Polymorphisms in the *APOA1/C3/A4/A5* cluster have been associated with triglyceride (TG) responses to fibrates [84]. Variants in this cluster, along with variants in LFABP, LIPC, ABCG8, and FABP1, collectively explain nearly 20% of the variation in lipid responses to fibrates [85]. Rare variant analyses have also identified additional variants in PPARα [86], LPL, and APOC-III [87] associated with TG and HDL-C (high-density lipoprotein cholesterol) responses to fibrate treatment. Genome-wide association studies conducted in the GOLDN and ACCORD trials identified variants in *PBX4* [88], *SMAD3*, and *IPO11* [89] associated with fibrate response. Rare variant analyses in the ACCORD trial revealed variants in *AKR7A3* [89] and *HSD17B13* [89] linked to fibrate response. Similarly, rare variant analyses in the GOLDN trial uncovered additional genetic hits in *ITGA7, SIPA1L2*, and *CEP72*, particularly associated with powerful responses to fibrates [90]. In another genome-wide association study, the polymorphism in *rs964184* locus located near the *APOA1* gene was identified as the most reliable predictor of lipid response to fenofibrate [91]. This locus demonstrated statistically significant associations with changes in HDL (high-density lipoprotein) cholesterol and triglyceride levels, and it nearly reached statistical significance for its effect on LDL (low-density lipoprotein) cholesterol levels. In conclusion, these findings, although promising, did not reach the stage of actionable results to date [92]. Extensive efforts toward validation and replication are necessary to further understand and leverage the genetic factors influencing fibrate responses.

## 4. Anticoagulant Drugs

VKAs and direct oral anticoagulants (DOACs) are used to treat and prevent thromboembolic disorders.

### 4.1. Warfarin

Warfarin, as a coumarin derivative, exerts its pharmacological activity by inhibiting VKOR, thereby blocking the synthesis of functional coagulation factors II, VII, IX, and X, as well as proteins C and S [93]. Warfarin in clinical use is a racemic combination with each of the two enantiomers having its own route of metabolism. The S enantiomer is converted into inactive metabolites by CYP2C9, whereas the R enantiomer is metabolized by cytochrome 1A1 (CYP1A1), cytochrome 1A2 (CYP1A2), and CYP3A4 [94]. S-warfarin has a significantly greater anticoagulant effect than R-warfarin. Because of warfarin’s limited therapeutic index, anticoagulation must be maintained within the international normalized ratio (INR) range of 2.0–3.0 in order to optimize efficacy while minimizing the risk of bleeding. To avoid mortality and morbidity from thromboembolic or bleeding events, coagulation must be closely monitored and doses must be adjusted frequently [95]. Although genotype is an important contributor to dosage variability, it is rarely considered in clinical practice. In spite of this, Warfarin was the first drug to have pharmacogenomics dosing guidelines. The most important genes regulating warfarin response are *CYP2C9* and *vitamin K epoxide reductase C1* (*VKORC1*), with *cytochrome 4F2* (*CYP4F2*) playing a minor role [96]. S-warfarin clearance, and thus patient dose requirements, are reduced by the CYP2C9*2, *3, *5, *6, *8, and *11 alleles. Patients with these genotypes will require lower doses and will be at a higher risk of serious bleeding complications. The *2 and *3 alleles are the most common among people of European ancestry, whereas the *5, *6, *8, and *11 alleles are mainly found in people of African ancestry [96]. Furthermore, 16 new polymorphisms in the CYP2C9 gene were discovered in Asian people, but their relevance in the warfarin dose adjustment is unknown [97]. VKORC1 is a warfarin protein target, and SNPs in this gene contribute to warfarin resistance and to the requirement for very high doses to achieve therapeutic anticoagulation [98]. The *VKORC1* regulatory region SNP -1639 G>A has been linked to lower VKORC1 expression and a clinically substantial reduction in warfarin dosages required. When compared to people of European ancestry, Asians have a higher prevalence of the -1639 AA (highly sensitive) genotype, while African individuals have a higher prelavence of the -1639 GG (reduced sensitivity) genotype. This variation accounts for the lower doses commonly reported in Asian individuals and higher doses observed in African individuals than in European individuals [99]. An SNP in *CYP4F2*, which catalyzes the conversion of free vitamin K to hydroxyvitamin K, increases warfarin dose requirements [99]. In European and Asian populations, the *3 allele is associated with higher warfarin dose requirements than the *1 allele [100]. However, no relationship has been observed among people of African ancestry [101]. The guidelines recommend the use of the IWPC algorithm to determine the warfarin dose to be used. The clinical and genetic information used in the algorithm is shown in Table S3 [102].

### 4.2. Heparin

When anti-coagulants are administered intravenously in emergency settings, such as heparins intravenously administered in the acute coronary syndrome, atrial fibrillation (AF), hemodialysis, or during cardiac surgery to prevent the formation of blood clots in devices used in extracorporeal circulation, it can be a challenge to assess the interindividual variability of response [103,104].

Various data in the literature describe known polymorphisms that influence the action of heparins, either making individuals resistant to their effect or increasing the incidence of major side effects such as heparin-induced thrombocytopenia (HIT). Patients with a high risk of HIT as determined by genotyping may receive intensive care or switch anticoagulant medications [105].

The same dose of unfractionated heparin (UFH) produces variable therapeutic responses in different patients, which can be monitored using activated clotting time (ACT). Abnormalities in antithrombin (AT) activity and mutation in its gene serpin family C member 1 (*SERPINC1*) can explain UFH resistance [106,107]. To date, more than 200 different mutations in SERPINC1 have been identified that are associated with lower levels of AT in addition to reduced anticoagulant activity [108]. AT deficiency is found in 0.02–0.05% of the general population and 0.5–5% of patients with venous thromboembolism (VTE), where it can be considered an important risk factor for morbidity [109,110].

The pharmacogenetics of heparins mainly concern the immunological side effect HIT, which emerges as thrombocytopenia and possibly life-threatening thrombosis. The identification of the several polymorphisms analyzed prior to heparin administration could aid in determining the group of patients at risk. *FcγRIIa-H131R* (*G507A*; *rs1801274*) and *FcγRIIIa-F158V* (*G559T*; *rs396991*) are the most investigated polymorphisms; however, there is no consistent evidence for their role in HIT [111,112].

### 4.3. Direct Oral Anticoagulants

DOACs have overcome the drawbacks of both VKAs and parenteral LWMHs, specifically, oral administration with no requirement for routine blood monitoring, strong therapeutic adherence, and fewer intracranial hemorrhages. Based on these data, DOACs are considered to be the first choice for anticoagulation therapy in various clinical circumstances [113,114,115,116,117], especially in patient categories under-represented in RCTs [63,118,119,120,121,122,123,124,125,126,127,128,129]. Genotyping could be used in this field not only to create dose schedules, but also to identify patients who would benefit from switching to DOACs. According to the Clinical Pharmacogenetics Implementation Consortium (CPIC) guidelines, oral anticoagulants may be recommended in patients who are poor metabolizers or have higher sensitivity.

#### 4.3.1. Dabigatran

Dabigatran etexilate is rapidly absorbed from the gut. As a prodrug, it must be converted into a pharmacologically active metabolite by intestinal carboxylesterase 2 and hepatic carboxylesterase 1 (CES1) to directly inhibit thrombin. The interindividual variability in metabolism generates variation in plasma concentration, which can result in a different response to treatment [105].

A genome-wide associations study (GWAS) of 2944 patients from the randomized evaluation of long term anticoagulant therapy (RE-LY) cohort found that three genotypes influenced dabigatran plasma levels; two were *CES1-associated* genotypes and one was an *ABCB1-associated* genotype [130]. Patients with the *CES1 rs8192935* polymorphism had a 12% reduction in peak plasma dabigatran concentrations, whereas the *ABCB1 rs4148738* and *rs1045642* polymorphism had an increase in dabigatran peak concentration without bleeding or ischemic events [130,131]. Another significant *CES1 SNP rs2244613* was linked to a 15% decrease in trough concentration and a lower risk of overall bleeding [130] without associated thromboembolic events [130,132].

However, a further study of 92 stable patients with atrial fibrillation found no significant association, except for a strong positive correlation between *CES1 SNP rs8192935* and reduced drug levels [113]. To date, genetic variations cannot be considered as a predictive marker for determining the optimal dose in patients with non-valvular AF treated with dabigatran [114]. In the same study, the researchers found that co-administration of dabigatran with a CYP3A4 inhibitor (e.g., clarithromycin) leads to inappropriately elevated plasma concentrations of dabigatran [115].

#### 4.3.2. Rivaroxaban

Concerning the *ABCB1* gene, a case of rivaroxaban-induced hemorrhage was reported in 2016 in a patient with *rs2032582* and *rs1045642* mutations [116], which was also confirmed by studies conducted by Xie et al. in 2018, showing increased peak concentrations for these mutated genotypes [117]. A previous study conducted in 2017 found no significant increase in rivaroxaban peak concentrations in a cohort of healthy volunteers [115]. In the 2018 Sennesael study, mutations in rs1128503, rs2032582, and rs4148738 were found in three patients with major bleeding associated with high residual blood concentration [118].

A study published in 2018 by Sychev et al. demonstrated that peak and trough concentrations of rivaroxaban are dependent on CYP3A4 activity [119]. Furthermore, several CYP3A4 polymorphisms are known to decrease its activity, such as CYP3A4*22/rs35599367 [63,120] or CYP3A4*17/rs4987161 [121]. Another study conducted in 2019 on 78 patients found no significant differences in peak rivaroxaban concentration between the *ABCB1-rs1045642/CYP3A4-rs35599367* and *ABCB1-rs4148738/CYP3A4-rs35599367* mutated haplotypes compared to their respective wild-type haplotypes [122].

#### 4.3.3. Apixaban

Several studies have been conducted on the pharmacogenomics of apixaban; the first study, conducted in 2016, suggested an association between the *rs4148738* mutation of *ABCB1* and an increase in peak apixaban concentration [123]. A study conducted by Ueshima in 2017 on a cohort of 44 Japanese patients treated with DOACs for non-valvular AF showed a significant increase in the residual concentration/dose ratio of apixaban with CYP3A5*1/*3 or *3/*3 (*rs776746*) and ATP binding cassette subfamily G member 2 (*ABCG2*) *421A>A* (*rs2231142*) genotypes [120] compared to CYP3A5*1/*1 and *ABCG2 421C>C* genotypes; the variants *1236C>T* (*rs1128503*), *2677G>T* (*rs2032582*), and *3435C>T* (*rs1045642*) of the *ABCB1* gene, however, did not impact this relationship [124]. In 2018, Kruykoy conducted a study on a sample of 17 Russian patients treated with apixaban, 10 mg daily, and showed no significant impact of *ABCB1 rs1045642* and *rs4148738* or *CYP3A5 rs776746* polymorphisms on the pharmacokinetics of apixaban [125]. A case report in 2019 reported significantly increased apixaban plasma concentrations 3 h (peak) and 12 h after an oral dose: 1100 ng/mL and 900 ng/mL, respectively. These levels were notably higher than the expected range (91 to 321 ng/mL at peak and 41 to 231 ng/mL after 12 h) in a woman with renal failure. The increase could be attributed to four polymorphisms: *ABCB1 rs2032582*, *rs1045642*, *CYP3A5 rs776746*, and *ABCG2 rs2231142* [126]. The latest study, conducted in 2020, on 358 Caucasian patients with AF demonstrated the significant increase in peak and trough blood levels of apixaban with the ABCG2 421C>A variant [127]. To date, no study has investigated the impact of mutations in the sulfotransferase 1A1 (*SULT1A1*) gene, whose SULT1A1*3 variant shows a moderate effect on the efficacy of apixaban [128] Therefore, as with other DOACs, genome-wide association analysis is required to confirm whether the effects of these polymorphisms are clinically significant.

#### 4.3.4. Edoxaban

To date, the evidence is based on a small pharmacokinetic cohort study that highlighted that the SNPs *rs4149056* in the *SLCO1B1* gene and *rs1045642* in the *ABCB1* gene do not modify the activity of edoxaban and have no clinical effects [129].

It is possible that the genes encoding *CES1* and *CYP3A4/5* involved in drug metabolism have clinical significance. However, as with other DOACs, further studies are still needed to create and implement pharmacogenetic-based treatment protocols.

#### 4.3.5. Betrixaban

Betrixaban is the most recently registered anticoagulant of the DOACs group. To date, no data are available on the genetic polymorphisms and on the pharmacokinetics and pharmacodynamics of betrixaban. Although, as with other DOACs, polymorphisms in the ABCB1 gene can be expected to impact plasma concentrations of the drug.

## 5. Conclusions

Several studies have clarified the genetic contributors to the inter-individual cardiovascular drug response variability. To date, there are recommendations about the detection of polymorphisms for starting or guiding the therapy with warfarin, clopidogrel, and statins. From a personalized medicine perspective, the genotypic information, when available, should be recorded in an electronic medical database and should be consulted in order to prescribe the best medical treatment according to a patient-centered approach. In this way, DGIs can be avoided, improving therapeutic adherence, pharmacodynamic effects, and clinical outcomes. Since cardiovascular diseases are still the leading cause of morbidity and mortality in the world, an increased understanding of the genetic contributors to cardiovascular drug response would lead to a reduction in healthcare costs for emergency room visits and hospitalizations and an extension of life expectancy.

## Data Availability

Not applicable.

## Supplementary Materials

The following supporting information can be downloaded at: https://www.mdpi.com/article/10.3390/genes14112057/s1, Table S1: Dosing recommendations for Clopidogrel (CPIC guidelines); Table S2: Dosing recommendations for Statins (CPIC guidelines); Table S3: Patient characteristics utilized in IWPC algorithm.
