# Supplementary material for: Pharmacogenomics of Cardiovascular Drugs for Atherothrombotic, Thromboembolic and Atherosclerotic Risk
Source: Genes (Basel). 2023 Nov 9;14(11):2057. doi: 10.3390/genes14112057 (PMC10671139; doi:10.3390/genes14112057)
Supplement: Supplementary file 1 [file genes-14-02057-s001.zip › genes-2667432-supplementary.pdf]

Table S1. Dosing recommendations for Clopidogrel (CPIC guidelines)[1]

Drug	Enzyme	Phenotype	Clinical implications	Reccomendation for therapy modification	CPIC guidance
Clopidogrel	CYP2C19	UM	Increased clopidogrel active metabolite formation<br>lower on- treatment platelet reactivity<br>no association with higher bleeding risk	Use standard dose (75 mg/day)	Strong
		RP	Normal or increased clopidogrel active metabolite formation<br>normal or lower on-treatment platelet reactivity<br>no association with higher bleeding risk	Use standard dose (75 mg/day)	Strong
		NM	Normal clopidogrel active metabolite formation<br>normal on-treatment platelet reactivity	Use standard dose (75 mg/day)	Strong
		IM	Reduced clopidogrel active metabolite formation<br>increased on-treatment platelet reactivity<br>increased risk for adverse cardiac and cerebrovascular events	Avoid Clopidogrel if possible; use Prasugrel or Ticagrelor at standard dose if no contraindication	Strong
		PM	Significantly reduced clopidogrel active metabolite formation<br>increased on-treatment platelet reactivity<br>increased risk for adverse cardiac and cerebrovascular events	Avoid Clopidogrel if possible; use Prasugrel or Ticagrelor at standard dose if no contraindication	Strong

UM: Ultrarapid Metabolizer; RP: Rapid Metabolizer; NM: Normal Metabolizer; IM: Intermediate Metabolizer; PM: Poor Metabolizer.

Table S2. Dosing recommendations for Statins (CPIC guidelines) [1]

Drug	Enzyme	Phenotype	Clinical implications	Reccomendation for therapy modification	CPIC guidance
Atorvastatin	SLCO1B1	PF	Increased atorvastatin exposure as compared with normal and decreased function, which may translate to increased myopathy risk	Prescribe ≤20 mg as a starting dose and adjust doses of atorvastatin based on disease-specific guidelines. If dose >20 mg is needed for desired efficacy, consider rosuvastatin or combination therapy	Moderate
Fluvastatin	SLCO1B1	PF	Increased fluvastatin exposure as compared with normal and decreased function; typical myopathy risk with doses ≤40 mg	Prescribe ≤40 mg per day as a starting dose and adjust doses of fluvastatin based on disease- specific guidelines. If patient is tolerating 40 mg per day but higher potency is needed, a higher dose (>40 mg) or an alternative statin (see **Figure 1** for recommendations for alternative statins) or combination therapy could be considered. Prescriber should be aware of possible increased risk for myopathy with fluvastatin especially with doses >40 mg per day	Moderate
	CYP2C9	PF	Increased fluvastatin exposure as compared with normal and intermediate metabolizer, which may translate to increased myopathy risk.	Prescribe ≤20 mg per day as a starting dose and adjust doses of fluvastatin based on disease-specific guidelines. If dose >20 mg needed for desired efficacy, consider an alternative statin or combination therapy	Moderate
Lovastatin	SLCO1B1	PF	Increased lovastatin acid exposure as compared with normal and decreased function, which may translate to increased myopathy risk	Prescribe an alternative statin depending on the desired potency	Moderate
Pravastatin	SLCO1B1	PF	Increased pravastatin statin exposure as compared with normal and decreased function; typical myopathy risk with doses ≤40 mg	Prescribe ≤40 mg as a starting dose and adjust doses of pravastatin based on disease-specific guidelines. If patient is tolerating 40-mg dose but higher potency is needed, a higher dose (>40 mg) or an alternative statin (see **Figure 1** for recommendations for alternative statins) or combination therapy could be considered. Prescriber should be aware of possible increased risk for myopathy especially with pravastatin doses >40 mg	Moderate

Rosuvastatin	SLCO1B1	PF	Increased rosuvastatin exposure as compared with normal function and decreased function; typical myopathy risk with doses ≤20 mg	Prescribe ≤20 mg as a starting dose and adjust doses of rosuvastatin based on disease-specific and population-specific guidelines If dose >20 mg needed for desired efficacy, consider combination therapy	Moderate
	ABCG2	PF	Increased rosuvastatin exposure compared with normal and decreased function; unknown myopathy risk; increased lipid-lowering effects	Prescribe ≤20 mg as a starting dose and adjust doses of rosuvastatin based on disease-specific and population-specific guidelines. If dose >20 mg needed for desired efficacy, consider an alternative statin or combination therapy	Moderate
Simvastatin	SLCO1B1	PF	Increased simvastatin acid exposure compared with normal and decreased function; highly increased myopathy risk	Prescribe an alternative statin depending on the desired potency	Strong

PF: Poor Function

Table S3. Patient characteristics utilized in IWPC algorithm [1]

**Age**
Height
Weight
VKORC1 genotype
CYP2C9 genotype
Race
Taking Enzyme Inducer
Taking Amiodarone
